# Not everyone is the same: Latent profile analysis of food addiction, personality traits and loneliness among young adults

**DOI:** 10.1192/j.eurpsy.2021.323

**Published:** 2021-08-13

**Authors:** E. Charzynska, P. Atroszko, A. Brytek-Matera

**Affiliations:** 1 Institute Of Psychology And Institute Of Pedagogy, University of Silesia, Katowice, Poland; 2 Institute Of Psychology, University of Gdansk, Gdansk, Poland; 3 Institute Of Psychology, University of Wroclaw, Wroclaw, Poland

**Keywords:** loneliness, latent profile analysis, food addiction, personality traits

## Abstract

**Introduction:**

Food addiction (FA) has been found to correlate with personality traits and psychosocial factors (Zhao et al., 2018). However, the vast majority of studies on this subject use the variable-oriented approach, which assumes that relationships between specific variables are identical in a given population (Collins & Lanza, 2010).

**Objectives:**

The main aim of this study was to assess the heterogeneity of young adults with respect to food addiction, personality traits (extraversion, conscientiousness and emotional stability) and loneliness. The secondary aim was to examine the relationships between profile membership and well-being.

**Methods:**

The sample consisted of 1,157 young adults (58.1% women). The Yale Food Addiction Scale, the Ten-Item Personality Inventory and the Short Loneliness Scale were used in the present study. Various aspects of well-being were included (e.g. quality of life). Latent Profile Analysis was performed twice: in the full sample, and in the subsample of individuals with increased FA (defined as z-score ≥ 1; n = 213).

**Results:**

Four profiles were identified both in the full sample and in the subsample. The best functioning was observed in individuals who scored high on extraversion and low on loneliness, despite their relatively high levels of FA. Young adults who scored high on FA and loneliness, and low on extraversion, conscientiousness and emotional stability, were more likely to have the worst functioning.

**Conclusions:**

Our findings suggest that using the person-oriented approach may expand our knowledge on the role of personality traits and psychosocial factors in determining the effects of FA on well-being.

**Disclosure:**

No significant relationships.

